# Intergenerational support and depressive symptoms in old age: The difference between urban and rural China

**DOI:** 10.3389/fpubh.2022.1007408

**Published:** 2022-11-16

**Authors:** Chenxi Wang, Zhengkui Liu, Tianyong Chen, Jinfeng Wang, Xin Zhang, Buxin Han

**Affiliations:** ^1^CAS Key Laboratory of Mental Health, Institute of Psychology, Beijing, China; ^2^Department of Psychology, University of Chinese Academy of Sciences, Beijing, China; ^3^School of Psychological and Cognitive Sciences and Beijing Key Laboratory of Behavior and Mental Health, Peking University, Beijing, China

**Keywords:** intergenerational support, depression, older adults, urban China, rural China

## Abstract

**Objectives:**

Intergenerational support is associated with fewer depressive symptoms in old age. Uneven development has resulted in huge urban–rural disparities in China, which could lead to different intergenerational relationships. The present study aimed to examine whether intergenerational support was associated with depressive symptoms differently among urban and rural Chinese older participants.

**Methods:**

A sample of 3,498 participants from nine pairs of urban subdistricts and rural villages were included in the present study. Depressive symptoms were measured by the 10-item Center for Epidemiological Studies Depression Scale, and the intergenerational support mechanisms (financial, instrumental, and emotional) were assessed with a self-designed questionnaire.

**Results:**

Significant areas by support effect for depressive symptoms indicated different associations between intergenerational financial and emotional support and depressive symptoms in urban and rural areas. Specifically, urban older participants receiving emotional support from adult children and rural older participants receiving financial support from adult children showed fewer depressive symptoms. In both areas, participants receiving instrumental support showed fewer depressive symptoms.

**Conclusion:**

Our study is the first to compare the urban–rural disparity in association between intergenerational support and depressive symptoms in a developing country, China. The results support modernization theories proposing weakened economic function but intensified emotional ties in societies with higher level of development. Communication-based intergenerational emotional support should be promoted in urban areas, and formal support systems should provide financial and instrumental support to the vulnerable rural older population.

## Introduction

Intergenerational support is a well-documented protective factor against depression in old age (1, 2), and this protective effect also holds true in China (3–5). During the last several decades, uneven development in China has led to great urban–rural disparities in economics, industries, living standard, and even social norms (6–8). Intergenerational relationship is closely linked to social development (9), and it is therefore intriguing whether intergenerational support and its association with depressive symptoms among older adults differ in the unevenly developed urban and rural areas in this developing country.

The most significant gap between urban and rural China is in economic development, with households' disposable income per capita in urban areas being 2.8 times the figure in rural areas through 1997 to 2015 (10). Urban areas have more secondary and tertiary industries, and urban residents enjoys better housing, education, and public services (8, 11). Furthermore, the social security system benefits urban older residents much more than their rural counterparts. The majority of rural older adults are covered by the Urban-Rural Resident Social Pension, the average of which being only 119 CNY per month at the time the present study was conducted, 5% of the Enterprise Employee Basic Pension, which covers most urban residents (12).

Such urban–rural disparities could lead to difference in intergenerational relationships. In agrarian societies with an economy based on land owned by the older generations, parents have authority over their children, and adult children are responsible for providing financial support to the older adults (13). In urban societies with a market economy, in contrast, the younger generation relies less on their parents to make a living, and the economic tie within a family is weakened (14, 15). At the same time, the improved pension system makes it possible for the older adults to support themselves (16). Consequently, adult children are no longer obliged to support their aged parents financially (17), and instead tend to maintain a strong emotional bond with their parents (18). Supporting this theory, empirical studies have found that in rural China, up to 60% of older adults receive financial support from their adult children (19, 20), while in developed countries (USA, European countries, and Japan) and urban China, the figure ranges from 1.3% to 22% (16, 19, 21, 22). Communication-based emotional support, on the other hand, is gaining importance and has become the leading form of intergenerational support expected by and provided to older adults in more developed areas such as Hong Kong, New Zealand, and Japan (22–24).

Intergenerational support has been found to be associated with few depressive symptoms in old age (25–28), but the contributions of different types of support are controversial. For example, with older participants from Myanmar, Vietnam, and Thailand, researchers found that financial but not emotional support from adult children predicted fewer depressive symptoms (27). In contrast, with participants from urban China, emotional but not financial support from adult children predicted fewer depressive symptoms in old age (4). Other studies in Japan and Spain found that both emotional and instrumental support from adult children was linked to fewer depressive symptoms (28, 29). These studies were conducted in areas with varied levels of social development, but it remains to be examined directly whether such difference has contributed to the inconsistent findings.

The present study aimed to test whether the association between three types of intergenerational support (financial, emotional, and instrumental) and depressive symptoms is different among urban and rural Chinese older participants. Basing on the above-described modernization theories and empirical studies, it was hypothesized that (1) in rural but not urban areas, older participants receiving financial support from adult children would show fewer depressive symptoms (H1), and (2) in urban but not rural areas, older participants receiving emotional support from adult children would show fewer depressive symptoms (H2). No hypothesis was made for instrumental support.

## Methods

### Sample and procedure

The present study is part of the Science and Technology Service Network Initiative and was initiated in 2015. The study was approved by the Ethics Committee of the Institute of Psychology of the Chinese Academy of Sciences (IPCAS), and informed consent was obtained from all participants.

**Figure 1:**
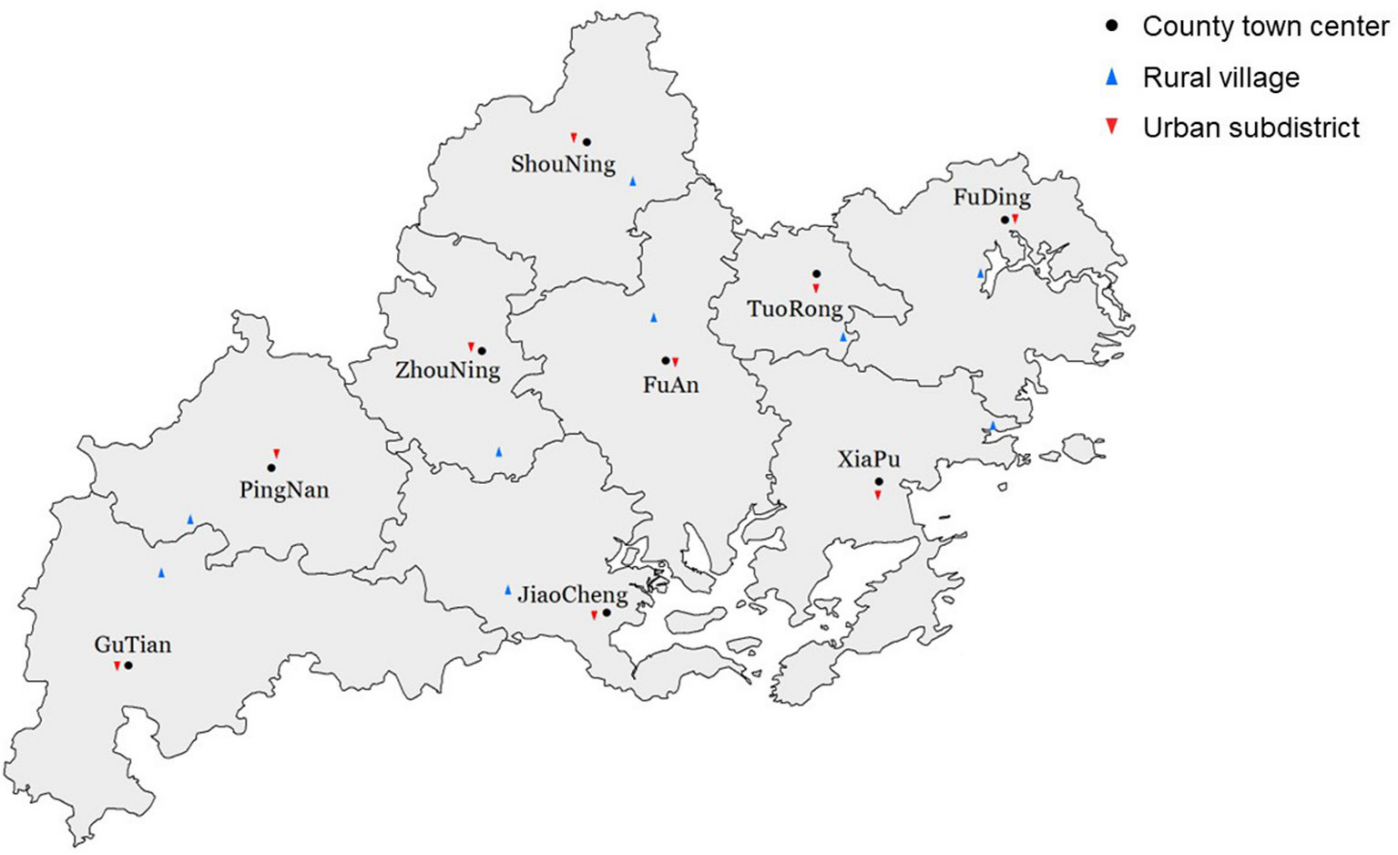
Sampling sites of the present study.

Data were collected from permanent resident older adults in Ningde, Fujian Province, and multistage sampling was used in the present study. At the first stage, purposive sampling was adopted to ensure differences in the sampling sites. From each of the nine county-level units in Ningde, one pair of rural village and urban subdistrict were chosen. The villages were far from the county town center, while the subdistricts were sampled in the towns (Figure 1). At the second stage, participants were sampled during an annual physical examination offered to all older adults (aged 60 or more). The physical examination lasted for a month and residents can take it on any day that they were available. For each site, on three pre-determined days during this period, older adults taking the physical examination on those days were consecutively enrolled until a sample size of 200 was reached.

Note that three or four decades ago, these rural and urban sampling sites were all traditional agricultural societies and shared similar agricultural economic structure and culture. However, because the latter type of sampling sites was close to the old town, they gradually evolved from the traditional agricultural villages to part of the towns due to urbanization. Therefore, this purposive sampling method provides us high-quality paired samples to compare the pattern of intergenerational support and its association with emotional well-being in old age at different levels of social development.

Data were collected through face-to-face interviews conducted by trained local healthcare workers using a structured questionnaire. The training of the interviewers consisted a key member training and a project training. Seven local healthcare workers with work experience in public health and health promotion participated the key member training, which was held at the IPCAS. Lectures on psychological well-being in old age were delivered, and the present study were introduced. The seven key members were responsible for coordinating the project, recruiting participants, and interviewing the participants. Another 15 local healthcare workers, together with the key members, participated in the project training, which was held at a local venue. Instructions on how to implement the interview were given by an experienced researcher. The healthcare workers discussed how to explain the items in local dialect, and practiced the interview through role playing. These 15 healthcare workers were responsible for interviewing the participants. The questionnaire contained 135 questions, and on average it took an hour for a participant to finish it.

A total of 3,620 participants completed the survey, of which 46 were excluded, being below the age of 60, and a further 76 were excluded due to missing data on one or more variables included in the regression models. The participants with missing data accounted for only 2.0% of all participants, and in both urban and rural areas, there was no significant difference in core variables (depressive symptoms and intergenerational support) between participants with or without missing data. Ultimately, data from 3,498 participants (1,675 from traditional villages and 1,823 from urban subdistricts) were used in analyses.

### Measures

#### Depressive symptoms

The 10-item version of the Center for Epidemiological Studies Depression Scale (CES-D) was used to measure depressive symptoms, requiring participants to indicate the frequency of depressive symptoms experienced during the past weeks on a 4-point scale from 0 (rarely or none) to 3 (most of the time) for each item. The total score ranges from 0 to 30, with higher scores indicating higher levels of depressive symptoms. The Chinese version of this scale has been validated in a previous study (30). The Cronbach alpha was 0.80 in the present sample.

#### Intergenerational support

The measurement of intergenerational support includes financial support, instrumental support, and emotional support. To assess financial support, participants were asked to identify at least one major source of income from the following options: (A) retirement pension, (B) spouse, (C) adult children, (D) adult grandchildren, (E) other relatives, (F) government subsidies, (G) labor income (working or farming), and (H) other sources. Answers were recoded into the variable “financial support from adult children.” Participants were coded as 1 if their choices included (C).

To measure instrumental and emotional support, participants were first asked to nominate up to three family members who provided the most support, then rate the support that each nominated child provided on a five-point Likert scale (1 = never to 5 = always), following the procedure of the Berlin Aging Study (31). The items of instrumental support were: (a) When you are sick, does he (or she) take care of you? (b) Does he (or she) help you to resolve difficulties? The items of emotional support are: (a) Do you confide in him (or her)? (b) Does he (or she) care for and comfort you? These questions were adapted from the Chinese Longitudinal Healthy Longevity Survey (32). For each item, the maximum among the scores corresponding to the nominees was used to represent the highest level of intergenerational support the participants receive. The sum of the two items' scores was used as the final score for instrumental/emotional support. Since the distribution of these two measurements were zero-inflated and significantly negatively skewed, both scores were dichotomized. A score of 8 or higher was coded as receiving “sufficient instrumental/emotional support.” This threshold value was chosen because a score of 8 means reporting “4-often” for both items or “5-always” for one item and “3-sometimes” for the other, which indicates intensive instrumental/emotional support.

#### Control variables

Demographic information (age, gender, marital status, living arrangement), socioeconomic status (education, personal income, perceived financial status), health condition (perceived health, physical function), and negative life events were assessed using a self-designed questionnaire.

Living arrangement was classified into three categories: with adult children (both with or without a spouse), with a spouse only, and alone. The variable “personal income” was recoded from the question “major source of income,” to indicate income other than intergenerational support. Participants who chose (A) retirement pension, (B) spouse, (E) other relatives, (G) labor income, or (H) other sources were coded as 1. Government subsidies were not included because every resident aged 60 and over is eligible for them, and they are hardly enough to maintain a minimum standard of living. Perceived financial status was measured by the question “Do you have enough money to spend?” Options ranged from “always” to “never.”

Perceived health was measured by a question asking participants to evaluate their health condition, with options ranging from “very good” to “very bad.” Activities of daily living (ADL) and instrumental ADL (IADL) were used to indicate physical functions. Activities of daily living assessed six items: bathing, dressing, toileting, indoor transferring, continence, and feeding. Instrumental activities of daily living assessed eight items: visiting others, shopping, cooking, doing laundry, walking for 2 km, carrying weights of 5 kg, squatting, and taking a bus. Participants having difficulty in neither ADL nor IADL were coded as “active,” those having difficulty only in IADLs as “mildly impaired,” and those having difficulty in both ADL and IADL as “severely impaired.” The negative life events variable assessed whether participants had experienced events such as bereavement or natural disaster during the past year.

### Statistical analysis

Independent sample *t*-tests and Chi-square tests were used to compare depressive symptoms with intergenerational support of participants in the two areas. We then conducted a one-way analysis of variance (one-way ANOVA) for bivariate analyses of associations between depressive symptoms and demographical variables, financial situation, and health situation.

Multiple linear regression models were then conducted for analyses of associations between depressive symptoms and intergenerational support. In the first set of models, interactions between area and intergenerational support were examined for their effect on depressive symptoms, both with and without other control variables. After having identified a significant interaction between area and intergenerational support, another set of models were conducted separately for urban and rural areas, examining the contribution of different types of intergenerational support on depressive symptoms.

## Results

### Descriptive analysis

Independent sample *t*-tests/chi-square tests were conducted on the mentioned demographics of participants from the two different areas. The results indicated that there was no significant difference in age distribution, marital status, or experience of negative life events (Table 1), but the percentage of females was higher in urban (56.6%) than in rural areas (50.7%, χ^2^ = 11.86, *p* < 0.001). Participants in the two areas differed significantly in living arrangements (χ^2^ = 75.32, *p* < 0.001), the percentage of participants living with adult children being higher in urban (55.3%) than in rural areas (42.3%). A larger proportion of participants in rural than in urban areas did not receive formal education (63.5% vs. 55.1%, χ^2^ = 106.61, *p* < 0.001), and were in a poor financial situation (49.2% vs. 34.3%, χ^2^ = 157.58, *p* < 0.001) and health condition (45.1% vs. 30.6%, χ^2^ = 90.79, *p* < 0.001). In rural areas, a lower percentage of participants reported retirement pensions as their major source of income than those in urban areas (5.7% vs. 23.3%, χ^2^ = 211.50, *p* < 0.001). Hence, these demographic variables were treated as covariates in the final regression models to rule out the possible influence of an unbalanced distribution.

**Table 1:** Sociodemographic characteristics of participants in the two areas.

	**Rural areas**	**Urban areas**		
	**(*n* = 1,675)**	**(*n* = 1,823)**		
**Variables**	***n* (%)**	***n* (%)**	**χ^2^**	***p***
Age			0.90	0.639
60–69	757 (45.2)	847 (46.5)		
70–79	636 (38.0)	688 (37.7)		
≥80	282 (16.8)	288 (15.8)		
Female	849 (50.7)	1,031 (56.6)	11.86	<0.001
Married	1,111 (66.3)	1,263 (69.3)	3.36	0.067
Living arrangement			75.32	<0.001
With children	709 (42.3)	1,008 (55.3)		
With spouse only	619 (37.0)	596 (32.7)		
Alone	347 (20.7)	219 (12.0)		
Formal education			106.61	<0.001
0 years	1,057 (63.1)	1,004 (55.1)		
1–6 years	519 (31.0)	506 (27.8)		
6+ years	99 (5.9)	313 (17.2)		
Financial situation			157.58	<0.001
Never or hardly enough	824 (49.2)	626 (34.3)		
Half the time enough	638 (38.1)	666 (36.5)		
Always or usually enough	213 (12.7)	531 (29.1)		
**Major personal income**				
Retirement pension	96 (5.7)	425 (23.3)	211.50	<0.001
Labor income	691 (41.3)	311 (17.1)	248.81	<0.001
Government subsidies	905 (54.0)	761 (41.7)	52.33	<0.001
Perceived health			90.79	<0.001
Bad or very bad	755 (45.1)	558 (30.6)		
Fair	712 (42.5)	891 (48.9)		
Good or very good	208 (12.4)	374 (20.5)		
Physical function (ADL/IADL)			25.64	<0.001
Active	1,264 (75.5)	1,487 (81.6)		
Mildly impaired	263 (15.7)	244 (13.4)		
Severely impaired	148 (8.8)	92 (5.0)		
Negative life events	342 (20.4)	366 (20.1)	0.04	0.835
**Intergenerational support**				
Financial support	1,057 (63.1)	1,030 (56.5)	15.82	<0.001
Emotional support	605 (36.1)	986 (54.1)	113.65	<0.001
Instrumental support	750 (44.8)	1,100 (60.3)	84.87	<0.001

### Depressive symptoms and intergenerational support in urban and rural areas

Participants in rural areas reported significantly higher levels of depressive symptoms (*M* = 12.30, *SD* = 5.88) than those in urban areas [*M* = 9.67, *SD* = 6.10, *t*_(3, 496)_ = 12.95, *p* < 0.001]. Intergenerational support also differed significantly in the two areas (Table 1). A higher percentage of participants in rural areas reported financial support from adult children as their major source of income (63.1% vs. 56.5%, χ^2^ = 15.82, *p* < 0.001). The reporting rate was significantly lower in rural than in urban areas for emotional (36.1% vs. 54.1%, χ^2^ = 113.65, *p* < 0.001) and instrumental support (44.8% vs. 60.3%, χ^2^ = 84.87, *p* < 0.001).

### Associations between depressive symptoms and control variables

Bivariate associations between depressive symptoms and control variables were then examined using the one-way ANOVA method (Table 2). In both urban and rural areas, higher levels of depressive symptoms were associated with increased age (*F* = 5.51, *p* < 0.01; *F* = 5.44, *p* < 0.01), being female (*F* = 16.40, *p* < 0.001; *F* = 10.06, *p* < 0.01), being unmarried (*F* = 21.92, *p* < 0.001; *F* = 11.01, *p* < 0.01), a poor financial situation (*F* = 70.14, *p* < 0.001; *F* = 130.57, *p* < 0.001), poor health (*F* = 103.22, *p* < 0.001; *F* = 154.74, *p* < 0.001), and negative life events (*F* = 60.38, *p* < 0.001; *F* = 130.89, *p* < 0.001).

**Table 2:** Bivariate analyses of association between depressive symptoms and related factors.

	**Rural areas (*****n*** = **1,675)**			**Urban areas (*****n*** = **1,823)**		
**Variables**	***M (SD)***	***F***	***p***	***M (SD)***	***F***	***p***
Age		5.51	0.004		5.44	0.004
60–69	12.05 (5.67)			9.62 (6.12)		
70–79	12.12 (5.93)			9.3 (5.86)		
≥80	13.35 (6.23)			10.7 (6.46)		
Gender		16.40	<0.001		10.06	0.002
Male	11.71 (5.76)			9.15 (6.06)		
Female	12.87 (5.95)			10.06 (6.1)		
Marriage		21.92	<0.001		11.01	0.001
Not currently married	13.23 (6.05)			10.38 (6.44)		
Currently married	11.82 (5.74)			9.35 (5.91)		
Living arrangement		7.84	<0.001		6.65	0.001
With children	12.26 (5.81)			9.4 (6.01)		
With spouse only	11.76 (5.58)			9.61 (5.86)		
Alone	13.31 (6.42)			11.05 (6.92)		
Formal education		25.92	<0.001		47.49	<0.001
0 years	12.99 (5.86)			10.74 (6.15)		
1–6 years	11.45 (5.84)			9.15 (5.79)		
6+ years	9.34 (4.8)			7.09 (5.5)		
Personal income		35.86	<0.001		123.93	<0.001
Yes	11.46 (5.81)			8.11 (5.89)		
No	13.16 (5.84)			11.19 (5.90)		
Financial situation		70.14	<0.001		130.57	<0.001
Never or hardly enough	13.82 (5.87)			12.46 (6)		
Half the time enough	11.34 (5.32)			9.04 (5.66)		
Always or usually enough	9.25 (5.77)			7.16 (5.39)		
Perceived health		103.22	<0.001		154.74	<0.001
Bad or very bad	14.08 (5.87)			12.6 (6.41)		
Fair	11.63 (5.33)			9.36 (5.39)		
Good or very good	8.1 (5.1)			6.03 (4.95)		
Physical function		43.257	<0.001		57.22	<0.001
Active	11.56 (5.71)			8.98 (5.67)		
Mildly impaired	14.23 (5.9)			12.31 (6.82)		
Severely impaired	15.12 (5.72)			13.83 (7.18)		
Negative life events		60.38	<0.001		130.89	<0.001
None	11.74 (5.63)			8.88 (5.64)		
At least one	14.46 (6.35)			12.82 (6.79)		

### Hypothesis testing

A series of multivariate regression analyses were conducted to examine whether the association between depressive symptoms and intergenerational support is different in the two areas (Table 3). In Model 1 (M1), only area, three types of intergenerational support, and interaction terms of the two variables were included, and control variables were added in Model 2 (M2). Regardless of this addition, the coefficients of interaction terms between area and financial support (M1, *B* = −1.94, *SE* = 0.42, *p* < 0.001; M2, *B* = −0.79, *SE* = 0.37, *p* < 0.05, H1) and area and emotional support (M1, *B* = 1.56, *SE* = 0.53, *p* < 0.01; M2, *B* = 1.16, *SE* = 0.47, *p* < 0.05, H2), reached statistical significance. This first set of models thus demonstrates that a difference exists in associations between depressive symptoms and financial/emotional support in urban and rural areas. Furthermore, in Model 1, the coefficients of main effects reached statistical significance for all three types of support (financial support, *B* = 1.96, *SE* = 0.28, *p* < 0.001; emotional support, *B* = −1.40, *SE* = 0.35, *p* < 0.001; instrumental support, *B* = −1.76, *SE* = 0.36, *p* < 0.001), and in Model 2, those do for emotional support (*B* = −1.02, *SE* = 0.31, *p* < 0.001) and instrumental support (*B* = −1.21, *SE* = 0.32, *p* < 0.001).

**Table 3:** Interaction of intergenerational support and area on depressive symptoms.

	**Model 1**			**Model 2**		
**Variables**	***B***	***SE***	**β**	***B***	***SE***	**β**
Rural areas	2.33	0.37	0.19***	1.16	0.33	0.09***
**Inter-generational support**						
Financial support	1.96	0.28	0.16***	−0.06	0.28	−0.00
Emotional support	−1.40	0.35	−0.11***	−1.02	0.31	−0.08**
Instrumental support	−1.76	0.36	−0.14***	−1.21	0.32	−0.10***
**Intergenerational support** *Area						
Financial support * Rural areas	−1.94	0.42	−0.15***	−0.79	0.37	−0.06*
Emotional support * Rural areas	1.56	0.53	0.10**	1.16	0.47	0.07*
Instrumental support * Rural areas	0.68	0.53	0.05	0.21	0.46	0.01
**Age (60–69)**						
70–79				−0.60	0.20	−0.05**
≥80				−0.11	0.28	−0.01
Female				0.67	0.19	0.05***
Married				−0.27	0.25	−0.02
**Living arrangement (Alone)**						
With children				−0.51	0.28	−0.04
With spouse only				−0.47	0.33	−0.04
**Education (0 years)**						
1–6 years				−0.56	0.21	−0.04**
6+ years				−1.29	0.31	−0.07***
Personal income				−1.54	0.22	−0.13***
**Financial situation (Always/usually enough)**						
Half the time enough				1.06	0.25	0.08***
Never or hardly enough				2.75	0.26	0.22***
**Perceived health (Good or very good)**						
Fair				2.50	0.26	0.20***
Bad or very bad				3.88	0.28	0.31***
**Physical function (Active)**						
Mildly impaired				1.57	0.26	0.09***
Severely impaired				2.64	0.35	0.11***
Negative life events				2.34	0.22	0.15***

^*^P < 0.05, ^**^P < 0.01, ^***^P < 0.001.

To further examine the difference in associations between depressive symptoms and intergenerational support, as well as other associated factors in urban and rural areas, another set of regression analyses were conducted for the two areas separately (Table 4). In Model 1 (M1) and Model 3 (M3), only three types of intergenerational support are included. In Model 2 (M2) and Model 4 (M4), control variables are also included. In models without covariates, financial support (H1) does not predict depressive symptoms in rural areas (*B* = 0.02, *SE* = 0.31, *p* > 0.05), and even predicts higher levels of depressive symptoms in urban areas (*B* = 1.96, *SE* = 0.28, *p* < 0.001). In models with covariates, financial support predicts lower levels of depressive symptoms in rural areas (*B* = −0.95, *SE* = 0.32, *p* < 0.01) and is no longer a significant predictor in urban areas (*B* = 0.05, *SE* = 0.30, *p* > 0.05). Emotional support (H2) predicts lower levels of depressive symptoms only in urban areas (M3, *B* = −1.40, *SE* = 0.35, *p* < 0.001; M4, *B* = −1.00, *SE* = 0.31, *p* < 0.01). Instrumental support, regardless of the variables considered, consistently predicts lower levels of depressive symptoms (M1, *B* = −1.08, *SE* = 0.39, *p* < 0.01; M2, *B* = −1.12, *SE* = 0.35, *p* < 0.01; M3, *B* = −1.76, *SE* = 0.36, *p* < 0.001; M4, *B* = −1.03, *SE* = 0.32, *p* < 0.001).

**Table 4:** Multiple liner regression models of intergenerational support on depressive symptoms in the two areas.

	**Rural areas**						**Urban areas**					
	**Model 1**			**Model 2**			**Model 3**			**Model 4**		
**Variables**	***B***	***SE***	**β**	***B***	***SE***	**β**	***B***	***SE***	**β**	***B***	***SE***	**β**
**Intergenerational support**												
Financial support	0.02	0.31	0.00	−0.95	0.32	−0.08**	1.96	0.28	0.16***	0.05	0.30	−0.00
Emotional support	0.15	0.40	0.01	0.19	0.36	0.02	−1.40	0.35	−0.12***	−1.00	0.31	−0.08**
Instrumental support	−1.08	0.39	−0.09**	−1.12	0.35	−0.10**	−1.76	0.36	−0.14***	−1.03	0.32	−0.08**
**Age (60–69)**												
70–79				−0.48	0.30	−0.04				−0.67	0.27	−0.05*
≥80				−0.04	0.41	0.00				−0.16	0.39	−0.01
Female				0.74	0.28	0.06**				0.59	0.26	0.05*
Married				−0.47	0.37	−0.04				−0.05	0.34	−0.00
**Living arrangement (Alone)**												
With children				−0.06	0.38	−0.01				−1.01	0.41	−0.08*
With spouse only				−0.45	0.45	−0.04				−0.58	0.48	−0.05
**Education (0 years)**												
1–6 years				−0.52	0.30	−0.04				−0.56	0.29	−0.04
6+ years				−1.80	0.57	−0.07**				−1.06	0.38	−0.07**
Personal income				−1.49	0.32	−0.13***				−1.56	0.31	−0.13***
**Financial situation (Always/usually enough)**												
Half the time enough				1.29	0.42	0.11**				0.87	0.31	0.07**
Never or hardly enough				2.79	0.42	0.24***				2.87	0.34	0.22***
**Perceived health (Good or very good)**												
Fair				2.66	0.42	0.22***				2.39	0.32	0.20***
Bad or very bad				4.03	0.43	0.34***				3.79	0.37	0.29***
**Physical function (Active)**												
Mildly impaired				1.45	0.37	0.09***				1.71	0.37	0.10***
Severely impaired				2.36	0.46	0.11***				3.06	0.56	0.11***
Negative life events				1.95	0.32	0.13***				2.74	0.31	0.18***

^*^P < 0.05, ^**^P < 0.01, ^***^P < 0.001.

It is important to note other indicators of depressive symptoms identified by this study. In both urban and rural areas, the three variables with the largest standard coefficients were poor perceived health (M2, *B* = 4.03, *SE* = 0.43, *p* < 0.001; M4, *B* = 3.79, *SE* = 0.37, *p* < 0.001), poor financial situation (M2, *B* = 2.79, *SE* = 0.42, *p* < 0.001; M4, *B* = 2.87, *SE* = 0.34, *p* < 0.001), and severely impaired physical function (M2, *B* = 2.36, *SE* = 0.46, *p* < 0.001; M4, *B* = 3.06, *SE* = 0.56, *p* < 0.001), indicating that these factors are most intensively associated with depressive symptoms.

## Discussion

The present study investigated whether the association between intergenerational support and depressive symptoms differed between urban and rural Chinese older adults. Given the important role intergenerational support plays in promoting emotional well-being in old age (1, 2), it is essential to find out which type of support benefits older parents more. Sociodemographic characteristics of the participants corroborated the difference of development in the two sets of sample sites. Participants in urban areas receive more formal education, rely more on retirement pension as their major source of income, and are in better financial situations, indicating different social and economic development levels of the two areas.

### Intergenerational support and depressive symptoms in old age

Consistent with the first hypothesis, results indicated that rural but not urban participants receiving financial support from adult children had fewer depressive symptoms, corroborating previous reports of contribution of financial support in rural China (33) and other developing countries (27). In addition, a higher percent of rural participants reported receiving intergenerational financial support than urban participants. In traditional Chinese societies, family is the most important source of old-age support, and adult children bear the responsibility to provide financial and material support for their aged parents (13). Older parents would feel ashamed and even consider it a personal failure in life if their children are unfilial and do not support them (34). However, financial support from adult children has gradually lost its psychological significance in societies with higher levels of development (23, 35, 36), as the role that a modern family plays as a productive unit declines (15, 37). Formal support systems have instead begun to address old-age security, reducing the dependence of older adults on their children, as is the case in urban China (38).

In the model for urban areas without covariates (Table 4, Model 3), participants relying on intergenerational financial support have more depressive symptoms. According to supplementary analyses, including the variable “personal income” in the model can lead to a change in the coefficient of financial support as in Model 4. We noticed that older adults without another income source are much more likely to depend on financial support from adult children than those with other incomes (85.2% vs. 28.1%, χ^2^ = 604.84, *p* < 0.001). Given that no other income is also a predictor of more depressive symptoms, it is reasonable that in Model 3, participants reporting intergenerational financial support, who were also less likely to have other incomes, had more depressive symptoms.

Consistent with the second hypothesis, urban but not rural participants receiving emotional support from adult children had fewer depressive symptoms, and a higher percent of urban participants reported receiving sufficient intergenerational emotional support than rural participants. This significance of emotional support from adult children corresponds with previous studies on older Chinese citizens (4, 39) and older adults (including Chinese immigrants) in developed countries (28, 29, 35). With economic function detached from modern families, intergenerational relationships are less structurally defined and more personal (18), and emotional ties are based more on “verbal communication, understanding, sharing thoughts, and showing feelings” (40). In the present study, items addressing emotional support focused on this intimacy-based emotional support, asking whether the participants “confide in” or “are cared for and comforted by” their adult children. In an East Asian traditional patriarchal society, however, where the parent-child relationship is asymmetric and obedience to parental authority is obligatory (41), mutual understanding was not valued. As the society modernizing, family ties still endure but are more likely driven by choice instead of obligation (42). Hence, in later life, emotional support from children contributes more to psychological well-being in urban areas.

It worth noting that less than half (44.8%) of the rural participants received sufficient intergenerational instrumental support, the figure significantly smaller than their urban counterparts. This lack of instrumental support in rural areas contradicts the obligation of adult children to provide old-age care to their parents, and it could be accounted for by the fewer proportion of participants living with adult children in rural (42.3%) than in urban (55.3%) areas. Traditionally, living with adult children is the most common and preferred living arrangement for older adults (43). Social development and changes in the last few decades, however, have shaken this tradition and led to an increase in nuclear families. In 2015, up to half of the older population do not live with adult children (44). Rural residents tend to seek more non-agricultural job opportunities and move to live in cities (45), leaving their older parents, while urban residents are more likely to work and live locally and hence, more possible to take care of their parents.

In both urban and rural areas, participants receiving sufficient instrumental support from adult children reported fewer depressive symptoms, consistent with previous nation-wide research (19). In China, with filial piety being an essential social norm and taking care of older parents prescribed by law, it is most prevalent that older adults live at home (23, 24, 46). Although the government has started to stimulate the expansion of residential care homes since this century, only <5% older adults living in such institutes, and existing institutes could not substitute care provided by adult children (47, 48). In addition, in modern societies, older adults need practical help from adult children with various additional things, including home repairs, transportation, and financial and legal matters (49). Under both social norms and legal requirements, intergenerational instrumental support still bears great significance in both urban and rural areas, and consequently, lacking intergenerational instrumental support could have contributed to the higher level of depressive symptoms among rural participants.

Furthermore, although it is not the primary concern, the present study identified other possible factors contributing to the great difference in depressive symptoms between urban and rural Chinese older adults. For participants in both areas, the predominant indicators of higher depressive levels are poor health, poor financial condition, and negative life events, all well-documented risk factors for late-life depression (50–53). The health and financial conditions of participants in rural areas are remarkably worse, which may have contributed most to the difference in late-life depressive symptoms in the two areas. This disparity is rooted in the unbalanced development in urban and rural China (7), rendering older adults in rural China the most vulnerable group in the context of mental health problems.

### Implications

The present study is the first to examine whether financial, emotional, and instrumental support from adult children is associated with depressive symptoms differently among urban and rural Chinese older adults. The results provided support for modernization theories proposing weakened economic function but intensified emotional tie in families in more developed societies (14, 18). To better meet the psychological needs of urban older adults, communication-based intergenerational emotional support should be promoted. Older adults in rural areas, on the other hand, are more vulnerable to depressive symptoms and need support from both formal and informal support systems. Economic development is fundamental to improving this situation; it can improve living conditions for rural older adults and create more local job opportunities, therefore alleviating rural-urban migration and the resulting lack of instrumental support from adult children. Further, formal support systems should provide better old-age security and life-care services to cover the basic needs of older residents in rural areas. These forms of formal support have started to be addressed (54).

### Limitations and future directions

The major limitation of this study is its generalizability. China is a vast nation with significant regional disparities in family values (55), and our sample sites were only from one prefecture-level city on the southeast coast of China. Other developing countries also differ from China in norms and practice concerning intergenerational relationships. Data from nation-wide surveys and other developing countries can further test whether the results can be generalized to other regions. Furthermore, it is intriguing whether other important formal (such as access to health care) and informal (such as friend) support systems influence urban–rural disparity of the association between intergenerational support and mental health. However, the contribution of other sources of support is beyond the scope of the present study and needs future studies to further investigate whether the present results still hold after controlling for other support systems.

## Data availability statement

The raw data supporting the conclusions of this article will be made available by the authors, without undue reservation.

## Ethics statement

The studies involving human participants were reviewed and approved by the Ethics Committee of the Institute of Psychology, Chinese Academy of Sciences. The patients/participants provided their written informed consent to participate in this study.

## Author contributions

CW performed all statistical analyses and contributed to writing the paper. ZL and JW contributed to survey implementation and data collection. TC planned the study, supervised the data analysis, and wrote the paper. XZ and BH contributed to revising the manuscript. All authors contributed to the article and approved the submitted version.

## Funding

This work was supported by the National Key Research and Development Program of China (2020YFC2003000); the Science and Technology Service Network Initiative (KFJ-EW-STS-025); and the Scientific Foundation of Institute of Psychology, Chinese Academy of Sciences (E2CX3715CX).

## Conflict of interest

The authors declare that the research was conducted in the absence of any commercial or financial relationships that could be construed as a potential conflict of interest.

## Publisher's note

All claims expressed in this article are solely those of the authors and do not necessarily represent those of their affiliated organizations, or those of the publisher, the editors and the reviewers. Any product that may be evaluated in this article, or claim that may be made by its manufacturer, is not guaranteed or endorsed by the publisher.

## References

[1] Schwarzbach M Luppa M Forstmeier S Konig HH Riedel-Heller SG. Social relations and depression in late life-a systematic review. Int J Geriatr Psychiatry. (2014) 29:1–21. 10.1002/gps.3971 23720299

[2] Wu M-H Chang S-M Chou F-H. Systematic literature review and meta-analysis of filial piety and depression in older people. J Transcult Nurs. (2018) 29:369–78. 10.1177/1043659617720266 29308707

[3] Li N Pang LH Chen G Song XM Zhang J Zheng XY. Risk factors for depression in older adults in Beijing. Can J Psychiat. (2011) 56:466–73. 10.1177/070674371105600804 21878157

[4] Liu J Guo M Mao W Xu L Huang X Chi I. Support from migrant children and depressive symptoms among Chinese older adults in transnational families. Gerontol Geriatr Med. (2018) 4:2333721418778187. 10.1177/2333721418778187 30035196 PMC6050608

[5] Wu Z Penning MJ. Children and the mental health of older adults in China: What matters? Popul Res Policy Rev. (2019) 38:27–52. 10.1007/s11113-018-9495-z

[6] Hu R Hu K. The compositional differences of social capital between rural and urban areas. J Xiamen Univ. (2008) 190:64–70. 10.3969/j.issn.0438-0460.2008.06.009

[7] Liu S Griffiths SM. From economic development to public health improvement: China faces equity challenges. Publ Health. (2011) 125:669–74. 10.1016/j.puhe.2011.08.004 21907369

[8] Treiman DJ. The “difference between heaven and earth”: urban–rural disparities in well-being in China. Res Soc Stratif Mobil. (2012) 30:33–47. 10.1016/j.rssm.2011.10.001

[9] Cherlin AJ. Goode's world revolution and family patterns: a reconsideration at fifty years. Popul Dev Rev. (2012) 38:577–607. 10.1111/j.1728-4457.2012.00528.x

[10] Wang X Shao S Li L. Agricultural inputs, urbanization, and urban-rural income disparity: evidence from China. China Econ Rev. (2019) 55:67–84. 10.1016/j.chieco.2019.03.009

[11] Qiao J. On the rural-urban disparity in access to higher education opportunities in China. Chin Educ Soc. (2010) 43:22–31. 10.2753/CED1061-1932430402

[12] Zhu H Walker A. Pension system reform in China: who gets what pensions? Soc Policy Admin. (2018) 52:1410–24. 10.1111/spol.12368

[13] Whyte MK. The persistence of family obligations in baoding. In:Whyte MK, editor. China's Revolutions and Intergenerational Relations. Ann Arbor, MI: University of Michigan Center for Chinese Studies (2003) pp. 85–120.

[14] Aboderin I. Modernisation and ageing theory revisited: current explanations of recent developing world and historical western shifts in material family support for older people. Ageing Soc. (2004) 24:29–50. 10.1017/S0144686X03001521

[15] Goode WJ. World Revolution and Family Patterns. New York, NY: Free Press (1963).

[16] Deindl C Brandt M. Financial support and practical help between older parents and their middle-aged children in Europe. Ageing Soc. (2011) 31:645–62. 10.1017/S0144686X10001212

[17] Caldwell JC. Toward a restatement of demographic transition theory. Popul Dev Rev. (1976) 2:321–66. 10.2307/1971615

[18] Šadl Z Hlebec V. Emotional support and intergenerational solidarity. Teorija Praksa. (2010) 47:1150–70. Retrieved from: http://dk.fdv.uni-lj.si/db/pdfs/tip20106_sadl_hlebec.pdf

[19] Mao X. The Relationship between Social Support and Subjective Well-Being Among Older Adults in China. Ph.D., New York University, Ann Arbor, MI (2018).

[20] Zheng X Liu L Pang L Qiu Y Yang C Chen Q. Effects of rapid economic development on traditional patterns of elder support in China. J Popul Ageing. (2012) 5:163–76. 10.1007/s12062-012-9065-9

[21] Cooney TM Dykstra PA. Family obligations and support behaviour: a United States–Netherlands comparison. Ageing Soc. (2011) 31:1026–50. 10.1017/S0144686X10001339

[22] Takagi E Saito Y. A longitudinal analysis of the impact of family support on the morale of older parents in Japan: does the parent's normative belief in filial responsibilities make a difference? Ageing Soc. (2013) 33:1053–76. 10.1017/S0144686X1200044X 23913993 PMC3728917

[23] Chong AML Liu S. Receive or give? Contemporary views among middle-aged and older Chinese adults on filial piety and well-being in Hong Kong Asia Pacific. J Soc Work Dev. (2016) 26:2–14. 10.1080/02185385.2015.1131619

[24] Li WW. Filial piety, parental piety and community piety: changing cultural practices of elder support among Chinese migrant families in New Zealand. OMNES J Multicult Soc. (2011) 2:1–30. 10.15685/omnes.2011.06.2.1.1

[25] Buber I Engelhardt H. Children's impact on the mental health of their older mothers and fathers: findings from the survey of health, ageing and retirement in Europe. Eur J Ageing. (2008) 5:31–45. 10.1007/s10433-008-0074-8 28798560 PMC5546383

[26] Li H Ji Y Chen T. The roles of different sources of social support on emotional well-being among Chinese elderly. PLoS ONE. (2014) 9:e90051. 10.1371/journal.pone.0090051 24594546 PMC3940715

[27] Teerawichitchainan B Pothisiri W Long GT. How do living arrangements and intergenerational support matter for psychological health of elderly parents? Evidence from Myanmar, Vietnam, and Thailand. Soc Sci Med. (2015) 136–7:106–16. 10.1016/j.socscimed.2015.05.019 25993521

[28] Tiedt AD. The gender gap in depressive symptoms among Japanese elders: evaluating social support and health as mediating factors. J Cross Cult Gerontol. (2010) 25:239–56. 10.1007/s10823-010-9122-x 20552391

[29] Zunzunegui MV Beland F Otero A. Support from children, living arrangements, self-rated health and depressive symptoms of older people in Spain. Int J Epidemiol. (2001) 30:1090–9. 10.1093/ije/30.5.1090 11689528

[30] Zhang J Wu Z Fnag G Li J Han B Chen Z. Development of the Chinese age norms of CES-D in urban area. Chin Ment Health J. (2010) 24:139–43. 10.3969/j.issn.1000-6729.2010.02.015

[31] Lang FR Schütze Y. Adult children's supportive behaviors and older parents' subjective well–being—a developmental perspective on intergenerational relationships. J Soc Issues. (2002) 58:661–80. 10.1111/1540-4560.00283

[32] Wang J Chen T Han B. Does co-residence with adult children associate with better psychological well-being among the oldest old in China? Aging Ment Health. (2014) 18:232–9. 10.1080/13607863.2013.837143 24053437 PMC4158609

[33] Cong Z Silverstein M. Intergenerational time-for-money exchanges in rural China: does reciprocity reduce depressive symptoms of older grandparents? Res Hum Dev. (2008) 5:6–25. 10.1080/15427600701853749

[34] Yang Y. Parental Dissatisfaction, Offspring's Filial Discrepancy, and Health and Wellbeing Among Older Chinese Adults. Ph.D., The University of Utah, Ann Arbor, MI (2016).

[35] Dong X Chang ES Wong E Simon M. A qualitative study of filial piety among community dwelling, Chinese, older adults: changing meaning and impact on health and well-being. J Intergener Relatsh. (2012) 10:131–46. 10.1080/15350770.2012.673393

[36] Herlofson K Hagestad G Slagsvold B Sørensen A-M. Intergenerational Family Responsibility and Solidarity in Europe. Multilinks Project Norwegian Social Research (NOVA) (104 Multilinks Project) (2011).30999801

[37] Beck U Beckgernsheim E. Individualization: Institutionalized Individualism and its Social and Political Consequences. London: Sage (2002).

[38] Wang D. China's urban and rural old age security system: challenges and options. China World Econ. (2006) 14:102–16. 10.1111/j.1749-124X.2006.00001.x

[39] Cheng S-T Chan ACM. Filial piety and psychological well-being in well older Chinese. J Gerontol B Psychol. (2006) 61:262–9. 10.1093/geronb/61.5.P262 16960229

[40] Jamieson L. Intimacy: Personal Relationships in Modern Societies. Cambridge: Polity Press (1998).

[41] Yeh K-H Yi C-C Tsao W-C Wan P-S. Filial piety in contemporary Chinese societies: a comparative study of Taiwan, Hong Kong, and China. Int Sociol. (2013) 28:277–96. 10.1177/0268580913484345

[42] Cai H Huang Z Jing Y. Living in a changing world: the change of culture and psychology. In:Matsumoto D Hwang HC, editors. The Handbook of Culture and Psychology. 2nd ed. New York, NY: Oxford University Press (2019) pp. 786–817.

[43] Yi Z Vaupel JW Zhenyu X Chunyuan Z Yuzhi L. Sociodemographic and health profiles of the oldest old in China. Popul Dev Rev. (2002) 28:251–73. 10.1111/j.1728-4457.2002.00251.x32603874

[44] Commission NHaFP. China Family Development Report 2015. Beijing: China Population Publishing House (2015).

[45] Yang XJ. China's rapid urbanization. Science. (2013) 342:310. 10.1126/science.342.6156.310-a 24136949

[46] Chow N. The practice of filial piety and its impact on long-term care policies for elderly people in Asian Chinese communities. Asian J Gerontol Geriatr. (2006) 1:31–5. Retrieved from: https://ecald.com/assets/Resources/Assets/Practice-Filial-Piety.pdf

[47] Shum MHY Lou VWQ He KZJ Chen CCH Wang J. The “leap forward” in nursing home development in urban China: future policy directions. J Am Med Dir Assoc. (2015) 16:784–9. 10.1016/j.jamda.2015.04.010 26027722

[48] Zhou Z Mao F Ma J Hao S Qian Z Elder K. A longitudinal analysis of the association between living arrangements and health among older adults in China. Res Aging. (2018) 40:72–97. 10.1177/0164027516680854 27932626

[49] Karpinska K Dykstra PA van den Broek T Djundeva M Abramowska-Kmon A Kotowska II. Intergenerational Linkages in the Family: The Organization of Caring and Financial Responsibilities: Summary of Results. Family and Societies Working Paper Series (2016). pp. 61.

[50] Beekman ATF Copeland J Prince MJ. Review of community prevalence of depression in later life. Brit J Psychiat. (1999) 174:307–11. 10.1192/bjp.174.4.307 10533549

[51] Cole MG Dendukuri N. Risk factors for depression among elderly community subjects: a systematic review and meta-analysis. Am J Psychiatry. (2003) 160:1147–56. 10.1176/appi.ajp.160.6.1147 12777274

[52] Fiske A Gatz M Pedersen NL. Depressive symptoms and aging: the effects of illness and non-health-related events. J Gerontol B. (2003) 58:320–8. 10.1093/geronb/58.6.P320 14614116

[53] Mirowsky J Ross CE. Age and depression. J Health Soc Behav. (1992) 33:187–205. 10.2307/2137349 1401846

[54] Williamson JB Fang L Calvo E. Rural pension reform in China: a critical analysis. J Aging Stud. (2017) 41:67–74. 10.1016/j.jaging.2017.04.003 28610757

[55] Hu Y Scott J. Family and gender values in China: generational, geographic, and gender differences. J Fam Issues. (2016) 37:1267–93. 10.1177/0192513x14528710

